# Supplementary material for: Content-rich biological network constructed by mining PubMed abstracts
Source: BMC Bioinformatics. 2004 Oct 8;5:147. doi: 10.1186/1471-2105-5-147 (PMC528731; doi:10.1186/1471-2105-5-147)
Supplement: Additional File 2 — The original results of the above study (non-essential files are deleted to keep the file size under the limit set by BMC bioinformatics). [file 1471-2105-5-147-S2.bz2 › chilibotAdditionalFile2/dip05/178ID9417082E616/html/PLAUR_FYN.html]

 


 **PLAUR** and **FYN** 
  
Found 4 abstracts in PubMed, retrieved 4.  
 

 What does Google say? 
 PDF only 
| .edu only 

---

**Interactive relationship** (e.g. stimulation, inhibition, etc)

**Neutral relationship**- We have found that anti uPAR  [ **PLAUR** ]  Ig precipitate the src type protein tyrosine kinases  **fyn** , hck and lck, which belong to a family of structurally and functionally related effectors participating in signalling from antigen and cytokine receptors.  Ref: 9654092 Eur J Biochem, 1998
